# Gamma Oryzanol Treats Obesity-Induced Kidney Injuries by Modulating the Adiponectin Receptor 2/PPAR-*α* Axis

**DOI:** 10.1155/2018/1278392

**Published:** 2018-09-09

**Authors:** Fabiane Valentini Francisqueti, Artur Junio Togneri Ferron, Fabiana Kurokawa Hasimoto, Pedro Henrique Rizzi Alves, Jéssica Leite Garcia, Klinsmann Carolo dos Santos, Fernando Moreto, Vanessa dos Santos Silva, Ana Lúcia A. Ferreira, Igor Otávio Minatel, Camila Renata Corrêa

**Affiliations:** ^1^Medical School, São Paulo State University (UNESP), Botucatu, SP, Brazil; ^2^Institute of Biosciences, São Paulo State University (UNESP), Botucatu, SP, Brazil

## Abstract

The kidney is an important organ in the maintenance of body homeostasis. Dietary compounds, reactive metabolites, obesity, and metabolic syndrome (MetS) can affect renal filtration and whole body homeostasis, increasing the risk of chronic kidney disease (CKD) development. Gamma oryzanol (*γ*Oz) is a compound with antioxidant and anti-inflammatory activity that has shown a positive action in the treatment of obesity and metabolic diseases. *Aim*. To evaluate the effect of *γ*Oz to recover renal function in obese animals by high sugar-fat diet by modulation of adiponectin receptor 2/PPAR-*α* axis *Methods*. Male Wistar rats were initially randomly divided into 2 experimental groups: control and high sugar-fat diet (HSF) for 20 weeks. When proteinuria was detected, HSF animals were allocated to receive *γ*Oz or maintain HSF for more than 10 weeks. The following were analyzed: nutritional and biochemical parameters, systolic blood pressure, and renal function. In the kidney, the following were evaluated: inflammation, oxidative stress, and protein expression by Western blot. *Results*. After 10 weeks of *γ*Oz treatment, *γ*Oz was effective to improve inflammation, increase antioxidant enzyme activities, increase the protein expression of adiponectin receptor 2 and PPAR-*α*, and recover renal function. *Conclusion*. These results permit us to confirm that *γ*Oz is able to modulate PPAR-*α* expression, inflammation, and oxidative stress pathways improving obesity-induced renal disease.

## 1. Introduction

Kidneys exert a central role in the maintenance of body homeostasis by regulating electrolyte concentrations, blood pressure, degradation of hormones, lipid metabolism, and excretion of waste metabolites [1]. Despite many factors leading to kidney disease, such as age, gender, smoking status, alcohol use, physical inactivity, diabetes mellitus, and hypertension, studies reveal that obesity is an independent risk factor for development of CKD [1–3].

The pathways activated by obesity to induce kidney disease are not fully understood. Studies have identified several new injurious pathways in the kidney led by insulin resistance (IR), chronic inflammation (a major contributor to microvascular remodeling), dyslipidemia and excessive nutrient availability (both may induce mitochondrial dysfunction and oxidative stress), and adipokine production unbalance [4–6].

Adiponectin is an adipocyte-derived protein hormone which plays a role in the suppression of inflammation-associated metabolic disorders. Adiponectin receptor 1 (Adipo-R1) and adiponectin receptor 2 (Adipo-R2) are the two major receptors for adiponectin and appear to be integral membrane proteins [7], expressed in different tissues, among them the kidney [8]. Kadowaki et al. [7] reported previously that the receptor expression levels are reduced in obesity, apparently in correlation with reduced adiponectin sensitivity. Moreover, the authors relate that Adipo-R1 may be more tightly linked to activation of AMPK pathways, whereas Adipo-R2 seems to be associated with the activation of PPAR-*α* pathways and the inhibition of inflammation. So, the modulation of these pathways could be important to treat renal injuries.

Considering this situation, natural compounds have received attention as a promising pool of substances to treat diseases [9]. Rice bran is rich in gamma oryzanol (*γ*Oz), a natural compound with antioxidant and anti-inflammatory activities that showed a positive action in the treatment of hyperlipidemia, hyperglycemia, insulin resistance, and increased levels of adiponectin [10–14]. So, considering that obesity, inflammation, and oxidative stress are able to induce renal disease and there are no studies that evaluate the effect of *γ*Oz in renal disease, the aim of this study was to evaluate the effect of *γ*Oz in the recovery of renal function in obese animals by high sugar-fat diet by modulation of the adiponectin receptor 2/PPAR-*α* axis.

## 2. Methods

### 2.1. Experimental Protocol

All of the experiments and procedures were approved by the Animal Ethics Committee of Botucatu Medical School (1150/2015) and were performed in accordance with the National Institute of Health's Guide for the Care and Use of Laboratory Animals. Male Wistar rats (±187 g) were kept in an environmental controlled room (22°C ± 3°C, 12 h light-dark cycle, and relative humidity of 60 ± 5%) and initially randomly divided into 2 experimental groups (control, *n* = 15, and high sugar-fat diet (HSF), *n* = 30) for 20 weeks. HSF groups also received water + sucrose (25%). The diets and water were provided ad libitum. The HSF diet contained soybean meal, sorghum, soybean peel, dextrin, sucrose, fructose, lard, vitamins, and minerals, plus 25% sucrose in drinking water; the control diet contained soybean meal, sorghum, soybean peel, dextrin, soy oil, vitamins, and minerals. The nutrients and nutritional composition of each diet was described in our previous study [15]. At week 20 of this study, when proteinuria was detected in the HSF groups, animals were divided to begin the treatment with *γ*Oz or continue receiving HSF for 10 more weeks as described below.

### 2.2. Group Characterization

After 20 weeks of experimental protocol, a 95% confidence interval (CI) was built for the protein/creatinine ratio from the HSF and control groups and was adopted as the separation point (SP) between the groups, the midpoint between the upper limit of the control group and the lower limit of the HSF group. The protein/creatinine ratio was adopted since it reflects proteinuria and is considered a marker of kidney function [16]. From this point, the control animals with a protein/creatinine ratio above of SP and the HSF animals with a protein/creatinine ratio below the SP were excluded from the control and HSF groups, respectively, ensuring the homogeneity of the treated and control groups. About the remaining animals in the HSF group, they were randomly divided to receive *γ*Oz or only diet. This criterion was adopted because animals submitted to different diet models do not always present the expected response. This fact can lead to erroneous animal classification and, consequently, false conclusions. The values for protein/creatinine ratio on the 20th week were 2.5 for the control group and 3.3 for the HSF group (*p* = 0.0006).

### 2.3. Treatment with Gamma Oryzanol

After the characterization on the 20th week, the groups were the following: control diet (control, *n* = 8), high sugar-fat diet (HSF, *n* = 8), and HSF/HSF + gamma oryzanol (HSF/HSF + *γ*Oz, *n* = 8). The treatment duration was 10 weeks, totaling 30 weeks of experiment. The *γ*Oz dose used in this study was added in the chow (0.5 *w*/*w*) according to our previous study [15].

### 2.4. Body Composition and Caloric Ingestion

The nutritional profile was evaluated according to the following parameters: caloric intake, body weight, and adiposity index. Caloric intake was determined by multiplying the energy value of each diet (g × kcal) by the daily food consumption. For the HSF group, caloric intake also included calories from water (0.25 × 4 × mL consumed). Body weight was measured weekly. After euthanasia, fat deposits (visceral (VAT), epididymal (EAT), and retroperitoneal (RAT)) were used to calculate the adiposity index (AI) by the following formula: [(VAT + EAT + RAT)/FBW] × 100.

### 2.5. Metabolic and Hormonal Analysis

After 12 h fasting, blood was collected and the plasma was used to measure insulin and biochemical parameters. Glucose concentration was determined by using a glucometer (Accu-Chek Performa, Roche Diagnostics Brazil Limited); triglycerides were measured with an automatic enzymatic analyzer system (Chemistry Analyzer BS-200, Mindray Medical International Limited, Shenzhen, China). The insulin and adiponectin levels were measured using enzyme-linked immunosorbent assay (ELISA) methods using commercial kits (EMD Millipore Corporation, Billerica, MA, USA). The homeostatic model of insulin resistance (HOMA-IR) was used as an insulin resistance index, calculated according to the following formula: HOMA-IR = (fasting glucose (mmol/L) × fasting insulin (*μ*U/mL))/22.5.

### 2.6. Systolic Blood Pressure

Systolic blood pressure (SBP) evaluation was assessed in conscious rats by the noninvasive tail-cuff method with a Narco Bio-Systems® electrosphygmomanometer (International Biomedical, Austin, TX, USA). The animals were kept in a wooden box (50 × 40 cm) between 38 and 40°C for 4-5 minutes to stimulate arterial vasodilation [17]. After this procedure, a cuff with a pneumatic pulse sensor was attached to the tail of each animal. The cuff was inflated to 200 mmHg pressure and subsequently deflated. The blood pressure values were recorded on a Gould RS 3200 polygraph (Gould Instrumental Valley View, Ohio, USA). The average of three pressure readings was recorded for each animal.

### 2.7. Renal Function

Renal function was evaluated by measurements of plasma and urine. At twenty-four hours, urine was collected from the metabolic cages to measure the excretion of creatinine and the total protein. The urea and creatinine content of the plasma were measured. All analyses were performed with an automatic enzymatic analyzer system (biochemical analyzer BS-200, Mindray, China). The glomerular filtration rate (GFR = (urine creatinine × flux)/plasma creatinine) and proteinuria were also calculated.

### 2.8. Renal Tissue Analysis

#### 2.8.1. Inflammatory Parameters

Renal tissue (±150 mg) was homogenized (ULTRA-TURRAX® T 25 basic IKA® Werke, Staufen, Germany) in 1.0 mL of phosphate-buffered saline (PBS) pH 7.4 cold solution and centrifuged at 800*g* at 4°C for 10 min. The supernatant (100 *μ*L) was used in analysis. Tumor necrosis factor-alpha (TNF-*α*), interleukin-6 (IL-6), and monocyte chemoattractant protein-1 (MCP-1) levels were measured using the enzyme-linked immunosorbent assay (ELISA) method using commercial kits from R&D System, Minneapolis, USA. The supernatant (100 *μ*L) was used for analysis, and the results were corrected by the protein amount.

### 2.8.2. Hydrophilic Antioxidant Capacity

The hydrophilic antioxidant capacity in the kidney was in the prepared supernatant as described in the previous item. It was determined fluorometrically, using a VICTOR X2 reader (PerkinElmer, Boston, MA). The antioxidant activity was quantitated by comparing the area under the curve relating to the oxidation kinetics of the suspension phosphatidylcholine (PC), which was used as the reference biological matrix. The peroxyl radical 2′,2′-azobis-(2-amidinopropane) dihydrochloride (AAPH) was used as an initiator of the reaction. The results represent the percent inhibition (4,4-difluoro-5-(4-phenyl 1-3 butadiene)-4-bora-3,4-diaza-s-indacene) (BODIPY) 581/591 plasma with respect to the control sample of BODIPY 581/591 PC liposome. All analyses were performed in triplicate. The results are reported as a percentage of protection [18].

### 2.8.3. Antioxidant Enzyme Activity

For these analyses, a 100 mg kidney was homogenized (1 : 10 *v*/*v*) in KH_2_PO_4_ (10 mmol/L)/KCl (120 mmol/L), pH 7.4, and centrifuged at 2.000 ×g for 20 min. Superoxide dismutase (SOD) activity was measured based on the inhibition of a superoxide radical reaction with pyrogallol, and the absorbance values were measured at 420 nm [19]. Catalase activity was evaluated by following the decrease in the levels of hydrogen peroxide in 240 nm [20]. The activity is expressed as pmol of H_2_O_2_ reduced/min/mg protein. Glutathione peroxidase (GP) activity was measured by following *β*-nicotinamide adenine dinucleotide phosphate (NADPH) oxidation at 340 nm as described by Flohé and Günzler [21]. The results were expressed as *μ*mol hydroperoxide-reduced/min/mg protein. Protein was quantified based on Lowry et al.'s method [22] using bovine serum albumin as the standard. The absorbance values for all analyses were measured in a UV/VIS spectrophotometer (Pharmacia Biotech, Houston, Texas, USA), and the values are expressed as units per milligram of protein.

### 2.8.4. Western Blot

Renal samples were homogenized in RIPA buffer with a protease and phosphatase cocktail inhibitor. After determination of protein concentration by the Bradford method [23], samples were diluted in Laemmli buffer and loaded (50 *μ*g of protein) into a 10% SDS–polyacrylamide gel. Transfer to a nitrocellulose membrane was carried out using Trans-Blot Turbo-Transfer System (BioRad). Incubation with the primary antibodies was performed overnight at 4°C in Tris-buffered saline solution containing Tween 20 (TBS-T) and 3% bovine serum albumin. Antibody dilutions were 1 : 1000 for Adipo-R1 (ABCAM ab126611), 1 : 1000 for Adipo-R2 (ABCAM ab77612), 1 : 500 for PPAR-*α* (ABCAM ab8934), 1 : 1000 for total AMPK (Cell Signaling #2532), 1 : 1000 for phospho-AMPH (Thr172) (Cell Signaling #2531), and 1 : 1000 for beta-actin (ABCAM ab8227). After incubation overnight at 4°C in TBS-T containing 1% nonfat dried milk with the Abcam secondary antibodies (dilution 1 : 3000 for anti-goat and 1 : 1000 for anti-rabbit). Protein was revealed using the chemiluminescence method according to the manufacturer's instructions (ECL SuperSignal® West Pico Chemiluminescent Substrate, Thermo Scientific). Band intensities were evaluated using ImageQuant TL 1D Version 8.1 (GE Healthcare Life Sciences).

### 2.9. Statistical Analysis

Data are presented as means ± standard deviation (SD) or median (interquartile range). Differences among the groups were determined by one-way analysis of variance. Statistically significant variables were subjected to the Tukey post hoc test to compare all the groups. Statistical analyses were performed using Sigma Stat for Windows Version 3.5 (Systat Software Inc., San Jose, CA, USA). A *p* value of 0.05 was considered statistically significant.

## 3. Results

**Figure 1:**
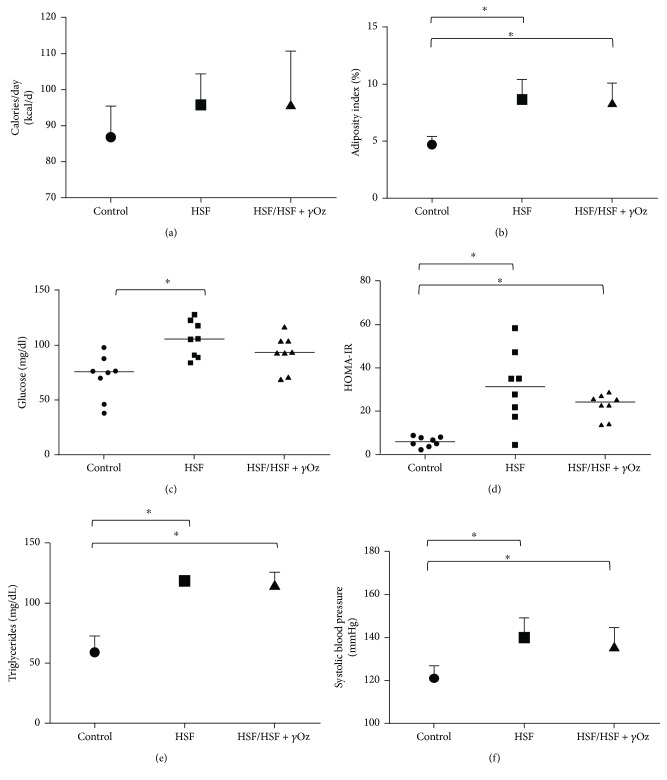
Nutritional, metabolic, and cardiovascular parameters: (a) caloric intake (kcal/day); (b) adiposity index (%); (c) glucose (mg/dL); (d) HOMA-IR; (e) triglycerides (mg/dL); (f) systolic blood pressure (mmHg). Data expressed in mean ± standard deviation or median. Comparison by one-way ANOVA with Tukey post hoc. HSF: high sugar-fat diet; *γ*Oz: gamma oryzanol. ∗ indicates *p* < 0.05; *n* = 8 animals/group.

Figure 1 shows caloric intake, adiposity index, and cardiometabolic risk factors for kidney disease (glucose, HOMA-IR, triglycerides, and systolic blood pressure). It is possible to verify that both HSF groups presented higher values for all the parameters. There was no difference for caloric intake.

**Figure 2:**
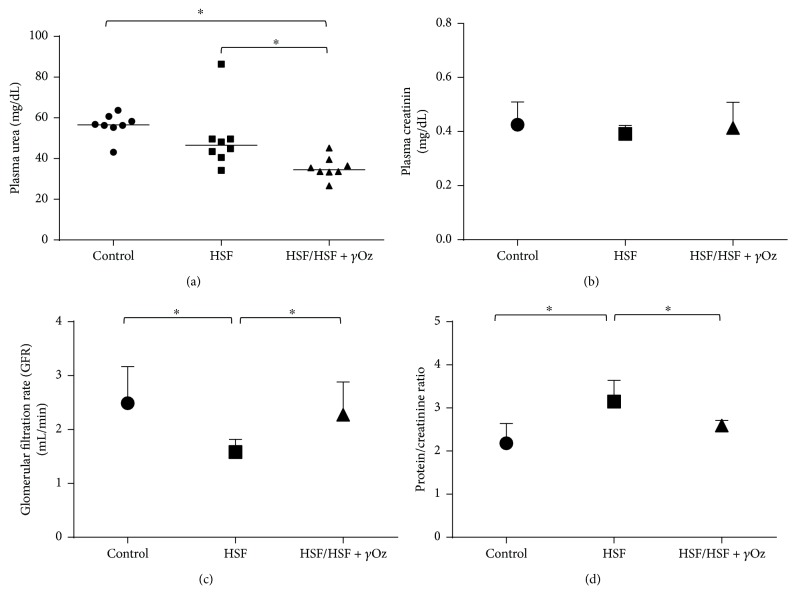
Renal function parameters: (a) plasma urea (mg/dL); (b) plasma creatinine (mg/dL); (c) glomerular filtration rate (GFR) (mL/min); (d) protein/creatinine ratio. Data expressed in mean ± standard deviation or median. Comparison by one-way ANOVA with Tukey post hoc. ∗ indicates *p* < 0.05; *n* = 8 animals/group.

Figure 2 shows renal function parameters. Gamma oryzanol was effective for recovery of renal function of the HSF/HSF + *γ*Oz group, characterized by lower proteinuria and high glomerular filtration rate compared to the HSF group.

**Figure 3:**
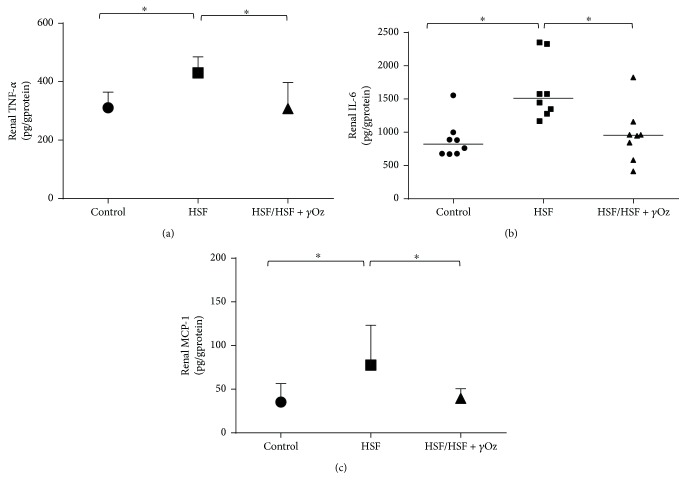
Inflammatory parameters in kidney tissue: (a) tumor necrosis factor-alpha (TNF-*α*; pg/g protein); (b) interleukin-6 (IL-6; pg/g protein); (c) monocyte chemoattractant protein-1 (MCP-1; pg/g protein). Data expressed in mean ± standard deviation or median. Comparison by one-way ANOVA with Tukey post hoc. ∗ indicates *p* < 0.05; *n* = 8 animals/group.

Figure 3 shows inflammatory parameters in kidney tissue. *γ*Oz was effective to reduce the inflammatory response for levels similar to those observed in the control group.

**Figure 4:**
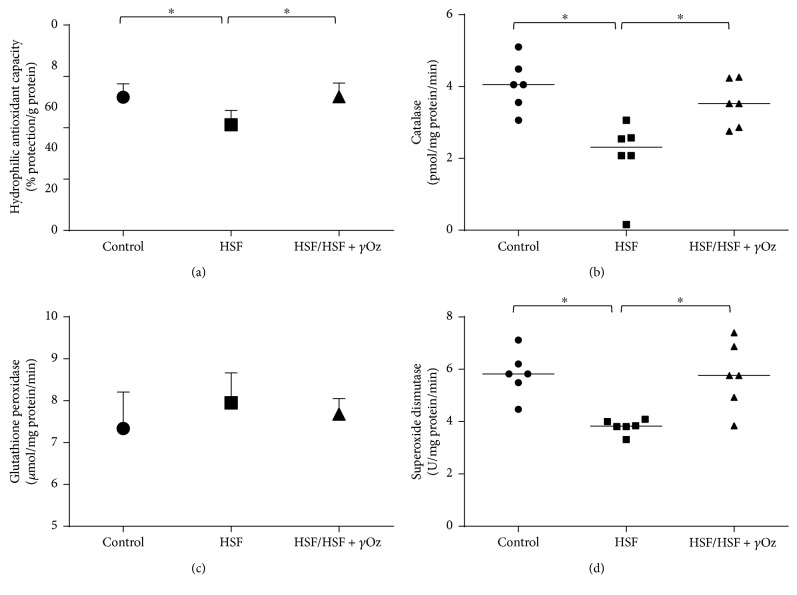
Redox state parameters in the kidney: (a) hydrophilic antioxidant capacity (% protection/g protein); (b) catalase (pmol/mg protein/min); (c) glutathione peroxidase (μmol/mg protein/min); (d) superoxide dismutase (U/mg protein/min). Data expressed in mean ± standard deviation. Comparison by one-way ANOVA with Tukey post hoc. ∗ indicates *p* < 0.05; *n* = 6 animals/group.

Figure 4 shows redox state parameters in the kidney. It is possible to verify a positive action of *γ*Oz on the HSF/HSF + *γ*Oz group to increase hydrophilic antioxidant protection, catalase, and superoxide dismutase levels compared to HSF.

**Figure 5:**
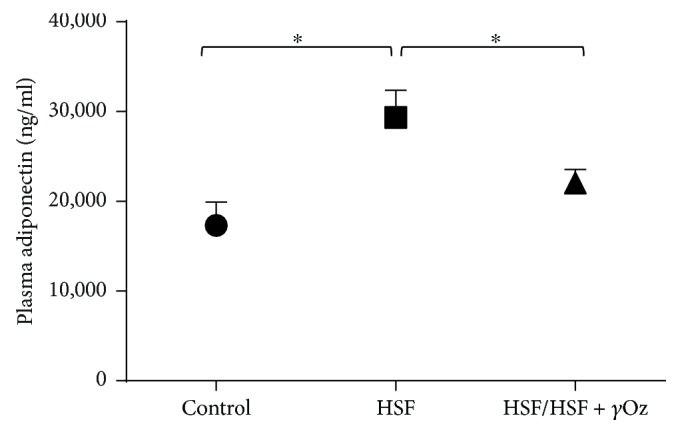
Plasma adiponectin levels (ng/mL). Data expressed in mean ± standard deviation. Comparison by one-way ANOVA with Tukey post hoc. ∗ indicates *p* < 0.05; *n* = 8 animals/group.

Figure 5 presents plasma adiponectin levels. The HSF group presented higher levels while the treatment with gamma oryzanol was able to reduce the levels.

**Figure 6:**
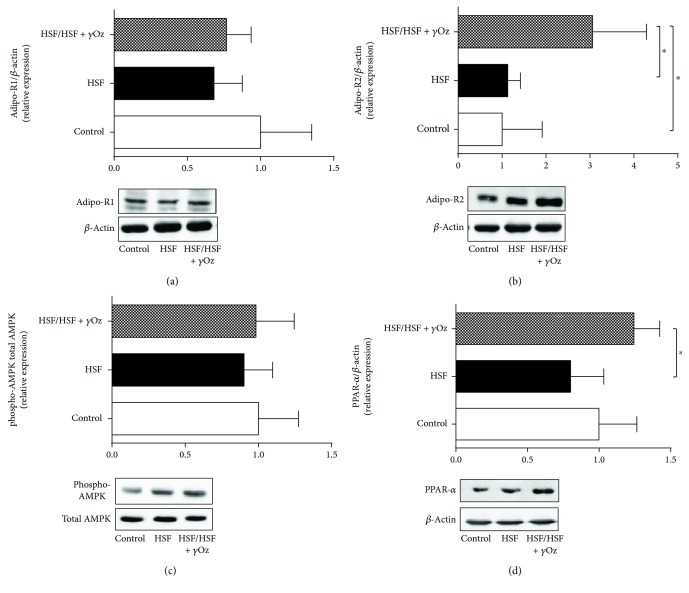
Relative protein expression in kidney tissue: (a) Adipo-R1; (b) Adipo-R2; (c) phospho-AMPK; (d) PPAR-*α*. Data expressed in mean ± standard deviation. Comparison by one-way ANOVA with Tukey post hoc. ∗ indicates *p* < 0.05; *n* = 6 animals/group.

Figure 6 shows protein expression of Adipo-R1, Adipo-R2, phosphorylated and total AMPK, and PPAR-*α* in the kidney. It is possible to note the effect of *γ*Oz which increased the expression of Adipo-R2 and PPAR-*α* when compared to HSF.

## 4. Discussion

The aim of this study was to evaluate the potential of *γ*Oz to recover renal function in obese animals by high sugar-fat diet consumption. In this study, the animals feeding on a HSF diet developed obesity and signals of kidney injury, characterized by proteinuria and decreased glomerular filtration rate. Obesity, insulin resistance, hypertension, chronic inflammation, dyslipidemia, and oxidative stress are considered the major risk factors for renal disease [1, 4, 6]. The HSF group developed all these risk factors, which were expected considering the diet used in this study, rich in sugar and fat [15], but the noneffect of *γ*Oz on these parameters was observed. In opposition to our results, Wang et al. and Justo et al. found in their studies improvement in some parameters after treatment with *γ*Oz [10, 24]. It is important to emphasize that in these studies, animal models and the dose of *γ*Oz were different from ours, which can explain these opposite results.

Once metabolic disorders are risk factors for renal disease, it would be expected that both HSF groups presented renal function impairment. However, analyzing the clinical signals of renal disease (proteinuria, most conveniently performed by estimation of the protein/creatinine ratio and glomerular filtration rate) [25], we can note an improvement in the treatment group with *γ*Oz characterized by lower proteinuria and higher GFR. Therefore, better understanding of the mechanisms by which *γ*Oz acted in this group is very important to enable novel therapeutic target development.

Oxidative stress is one condition associated with impaired renal function [8, 26]. Kidney disease progression is related with a significant increase of ROS, which influences cell function and damages proteins, lipids, and nucleic acids, and can also inhibit enzymatic activities of the cellular respiratory chairs. On the other hand, endogenous enzymatic and nonenzymatic antioxidant mechanisms protect against damaging effects of oxidative products [27]. The first line of enzymatic antioxidant defense is SOD, which accelerates the dismutation rate of oxygen to H_2_O_2_, but the catalase reduces H_2_O_2_ to water. Glutathione peroxidase reduces H_2_O_2_ and other organic peroxides to water and oxygen and requires glutathione as a hydrogen donor which is a scavenger for H_2_O_2_, hydroxyl radicals, and chlorinated oxidants [27]. Usually, patients suffering from renal insufficiency have diminished antioxidant defense when compared to healthy controls [28]. In the case of this study, the results showed an increase of antioxidant capacity, SOD, and catalase activities after treatment with gamma oryzanol, confirming the potential of the compound to improve the antioxidant system. But some authors relate difficulty in establishing a pattern of antioxidant status in kidney disease due to assessment by different measurement techniques [28]. In this case, information associating various parameters can give a better representation of a patient's current antioxidant status.

The literature reports that the renoprotection can also be related to some mechanisms involving improvement of the endothelial dysfunction, reduction of oxidative stress, and upregulation of endothelial nitric oxide synthase expression, all effects dependent on adiponectin receptor activation [29]. In contrast, the dysfunction regulation of adiponectin and its receptors has been observed in the development of various diseases, including obesity, insulin resistance, type 1 and type 2 diabetes, and chronic kidney disease [29].

Adiponectin is secreted primarily by adipose tissue and plays a key role in kidney disease. In obesity, reduced adiponectin levels are also associated with insulin resistance and cardiovascular disease. However, in conditions of established chronic kidney disease, adiponectin levels are elevated and positively predict progression of disease [30, 31]. Corroborating these findings, the HSF group presented higher levels of adiponectin associated with reduced GFR which confirms kidney disease. In opposition, the HSF group that received the compound showed reduction in the levels, which can be explained by the amelioration of glomerular filtration rate by *γ*Oz in these animals, since adiponectin is excreted via kidney glomerular filtration [32].

Adipo-R1 and Adipo-R2 are expressed in many tissues [8], but in the specific case of the kidneys, no studies evaluated the effect of *γ*Oz in this pathway and its role on renal function. The compound showed capacity to upregulate the Adipo-R2/PPAR-*α* axis. PPAR-*α* is highly expressed in tissues that possess high mitochondrial and *β*-oxidation activity, as the kidney. Decreased renal PPAR-*α* expression might contribute to the pathogenesis of kidney injuries [33], whereas its high expression is associated with metabolic control in the organ [34]. Moreover, PPAR-*α* activation can attenuate or inhibit several mediators of vascular injury involved in renal damage, such as lipotoxicity, reactive species oxygen (ROS) generation, and inflammation [34, 35]. Corroborating this information, our animals of the HSF/HSF + *γ*Oz group did not present inflammation in the kidney, showing lower levels of TNF-*α*, IL-6, and MCP-1 compared to the HSF group.

In summary, this study introduces very important findings since *γ*Oz was effective in ameliorating renal dysfunction by acting on the Adipo-R2/PPAR-*α* axis and also by improving the antioxidant response in the organ. *γ*Oz could be a therapeutic alternative for restoring/ameliorating metabolic dysfunctions, in special renal injuries that are developed in an obese individual. These results permit us to confirm that *γ*Oz is able to modulate PPAR-*α* expression, inflammation, and oxidative stress pathways improving obesity-induced renal disease.

## Data Availability

The data used to support the findings of this study are available from the corresponding author upon request.
